# Development of artificial intelligence prognostic model for surgically resected non-small cell lung cancer

**DOI:** 10.1038/s41598-023-42964-8

**Published:** 2023-09-21

**Authors:** Fumihiko Kinoshita, Tomoyoshi Takenaka, Takanori Yamashita, Koutarou Matsumoto, Yuka Oku, Yuki Ono, Sho Wakasu, Naoki Haratake, Tetsuzo Tagawa, Naoki Nakashima, Masaki Mori

**Affiliations:** 1 Department of Surgery and Science, Graduate School of Medical Sciences, Kyushu University, 3-1-1 Maidashi, Higashi-Ku, Fukuoka, 812-8582 Japan; 2 Department of Thoracic Oncology, National Hospital Organization Kyushu Cancer Center, Fukuoka, Japan; 3 Medical Information Center, Kyushu University Hospital, Fukuoka, Japan; 4 Biostastics Center, Kurume University, Fukuoka, Japan

**Keywords:** Cancer, Lung cancer

## Abstract

There are great expectations for artificial intelligence (AI) in medicine. We aimed to develop an AI prognostic model for surgically resected non-small cell lung cancer (NSCLC). This study enrolled 1049 patients with pathological stage I–IIIA surgically resected NSCLC at Kyushu University. We set 17 clinicopathological factors and 30 preoperative and 22 postoperative blood test results as explanatory variables. Disease-free survival (DFS), overall survival (OS), and cancer-specific survival (CSS) were set as objective variables. The eXtreme Gradient Boosting (XGBoost) was used as the machine learning algorithm. The median age was 69 (23–89) years, and 605 patients (57.7%) were male. The numbers of patients with pathological stage IA, IB, IIA, IIB, and IIIA were 553 (52.7%), 223 (21.4%), 100 (9.5%), 55 (5.3%), and 118 (11.2%), respectively. The 5-year DFS, OS, and CSS rates were 71.0%, 82.8%, and 88.7%, respectively. Our AI prognostic model showed that the areas under the curve of the receiver operating characteristic curves of DFS, OS, and CSS at 5 years were 0.890, 0.926, and 0.960, respectively. The AI prognostic model using XGBoost showed good prediction accuracy and provided accurate predictive probability of postoperative prognosis of NSCLC.

## Introduction

Lung cancer is one of the major causes of cancer-related death worldwide^1, 2^. Non-small cell lung cancer (NSCLC) is the most common type of lung cancer^3^, and surgical resection is one of the standard therapies along with chemotherapy and radiotherapy for NSCLC^4^. Tumor stage according to TNM classification is most commonly used to predict patient prognosis after surgery for NSCLC, and treatment strategy including postoperative adjuvant chemotherapy is determined based on the stage^4–6^. However, there are considerable differences in the prognosis of patients, even within the same stage. Therefore, better prognostic tools are required to more accurately determine patient prognosis and select the appropriate treatment strategy.

A number of studies have reported that various immune-nutrition indices calculated from blood test results, such as controlling nutritional status^7, 8^, geriatric nutritional risk index^9, 10^, prognostic nutritional index^11^, Glasgow prognostic score^12^, C-reactive protein (CRP)/albumin ratio^13, 14^, neutrophil/lymphocyte ratio^15^, platelet/lymphocyte ratio^15^, and monocyte/lymphocyte ratio^15, 16^, are prognostic factors for postoperative prognosis in NSCLC. However, most of these studies examined only a single factor or at most five factors, and there is still no consensus on the prognostic value of blood test results in NSCLC.

Artificial intelligence (AI) has shown great promise for important applications in medicine. In lung cancer research, several studies showed that AI is useful for the diagnosis^17–22^ and prediction of the therapeutic efficacy of chemotherapy^23^. In this study, we developed an AI prognostic model for NSCLC using machine learning to comprehensively analyze perioperative data including preoperative and postoperative blood test results.

## Results

### Patient characteristics

A total of 1,049 patients with pathological stage (p-Stage) I–IIIA NSCLC were enrolled. The clinicopathological characteristics of patients are shown in Supplementary Table 1. The median patient age at surgery was 69 (23–89) years, and 605 patients (57.7%) were male. The median body mass index and pack year index (PYI) were 22.4 (14.0–34.3) kg/m^2^ and 20 (0–300), respectively. In terms of surgical procedure, 144 (13.7%), 109 (10.4%), 752 (71.7%), 24 (2.3%), and 20 (1.9%) patients underwent wedge resection, segmentectomy, lobectomy, bilobectomy, and pneumonectomy, respectively. The numbers of patients with p-Stage IA, IB, IIA, IIB, and IIIA NSCLC were 553 (52.7%), 223 (21.4%), 100 (9.5%), 55 (5.3%), and 118 (11.2%), respectively. Regarding histological type, 151 (14.4%), 574 (54.7%), 55 (5.2%), 31 (3.0%), 189 (18.0%), 16 (81.5%), 6 (0.6%), 23 (2.2%), and 4 (0.4%) patients were diagnosed with adenocarcinoma in situ/minimally invasive adenocarcinoma/lepidic predominant adenocarcinoma, acinar/papillary predominant adenocarcinoma, micropapillary/solid predominant adenocarcinoma, squamous cell carcinoma, adenosquamous carcinoma, carcinoid, large cell neuroendocrine carcinoma, and pleomorphic carcinoma, respectively.

**Figure 1:**
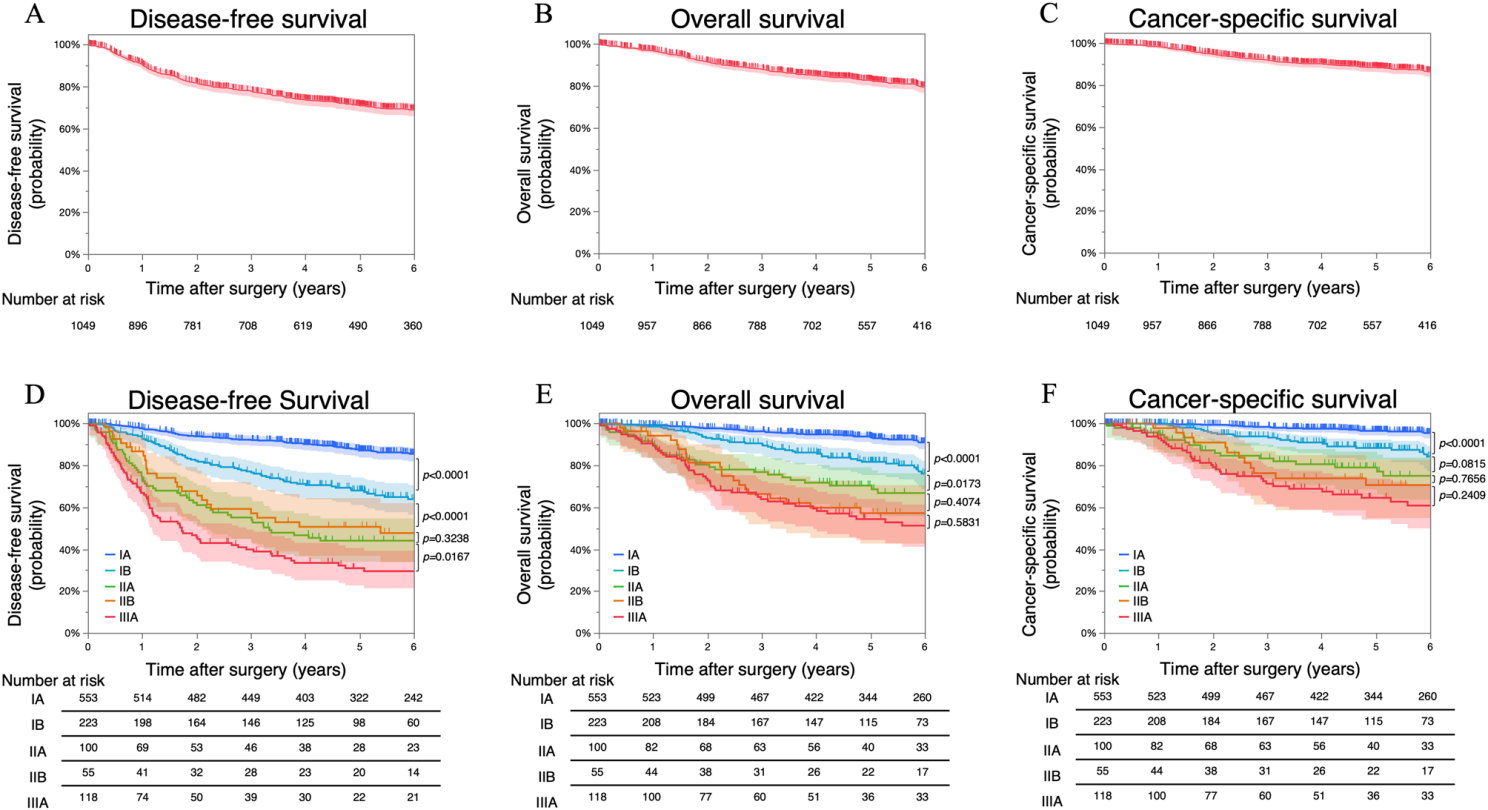
Survival curves plotted by the Kaplan–Meier method. The survival curves for disease-free survival (**A**), overall survival (**B**), and cancer-specific survival (**C**) in all patients. The survival curves of disease-free survival (**D**), overall survival (**E**), and cancer-specific survival (**F**) of patients according to pathological stage. The 95% confidence intervals are shown via shading.

The median follow-up period was 5.06 (0.02–17.39) years, and the number of disease-free survival (DFS), overall survival (OS), and cancer-specific survival (CSS) events were 305, 214, and 123, respectively. The survival curves of DFS (Fig. 1A), OS (Fig. 1B), and CSS (Fig. 1C) in the overall patient group were plotted by the Kaplan–Meier method. The 5-year DFS, OS, and CSS rates were 71.0%, 82.8%, and 88.7%, respectively. We also used the Kaplan–Meier method to plot survival curves of DFS (Fig. 1D), OS (Fig. 1E), and CSS (Fig. 1F) in patients according to p-Stage. In patients with p-Stage IA, IB, IIA, IIB, and IIIA NSCLC, the 5-year DFS rates were 87.1%, 68.0%, 44.0%, 50.7%, and 29.5%, respectively (log-rank test; IA versus IB, *p* < 0.0001; IB versus IIA, *p* < 0.0001; IIA versus IIB, *p* = 0.3238; IIB versus IIIA, *p* = 0.0167); the 5-year OS rates were 93.5%, 81.7%, 68.6%, 57.2%, and 54.4%, respectively (log-rank test; IA versus IB, *p* < 0.0001; IB versus IIA, *p* = 0.0173; IIA versus IIB, *p* = 0.4074; IIB versus IIIA, *p* = 0.5831); and the 5-year CSS rates were 96.5%, 88.3%, 77.1%, 70.6%, and 64.5%, respectively (log-rank test; IA versus IB, *p* < 0.0001; IB versus IIA, *p* = 0.0815; IIA versus IIB, *p* = 0.7656; IIB versus IIIA, *p* = 0.2409).

The results of univariable analyses of representative factors for DFS, OS, and CSS are shown in Supplementary Table 3, 4 and 5.

### Development of AI prognostic model

**Figure 2:**
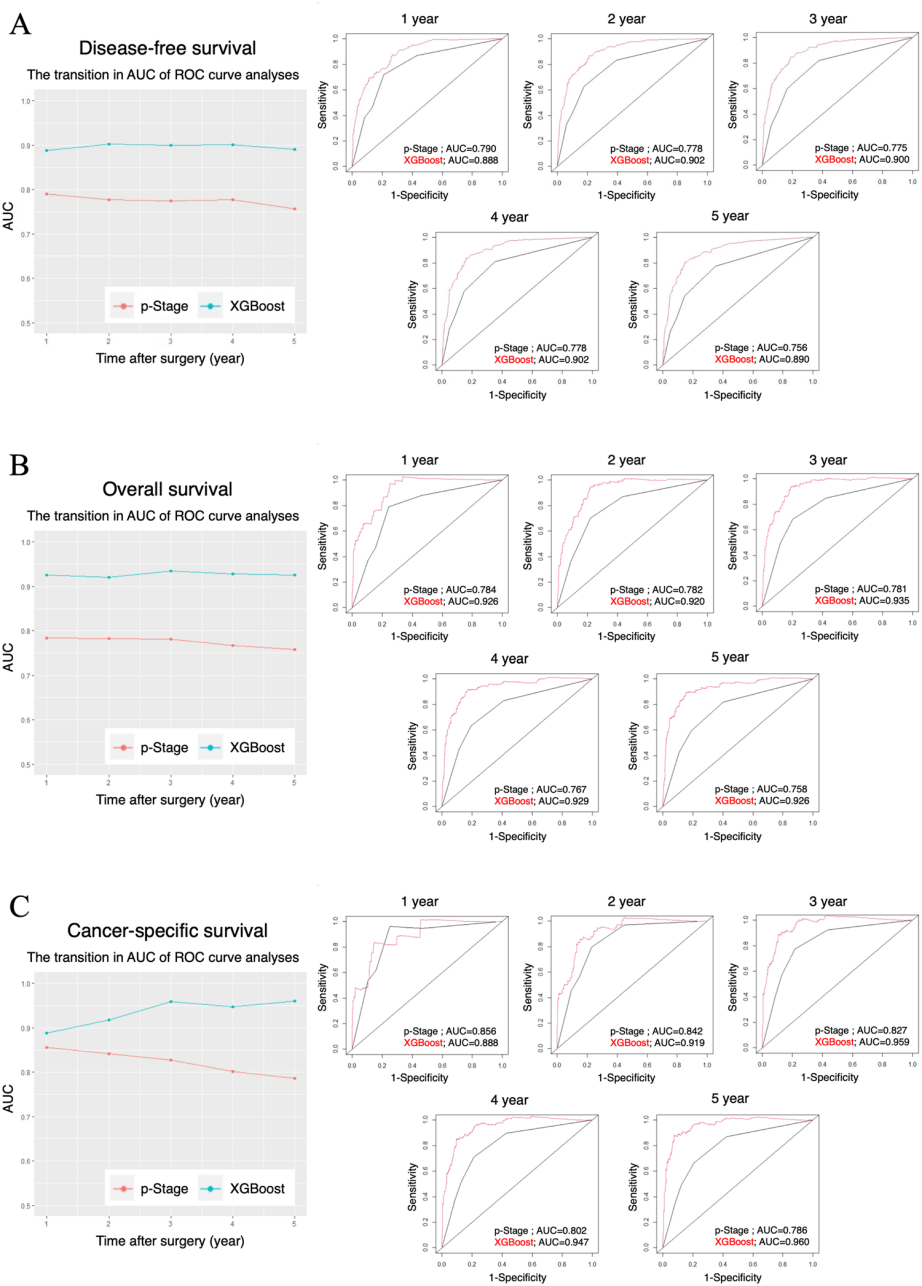
Time-dependent AUCs and ROC curves of the AI prognostic model. The transition in AUC over time and ROC curves at each point for disease-free survival (**A**), overall survival (**B**), and cancer-specific survival (**C**). AUC; area under curve, ROC; receiver operating characteristic, AI; artificial intelligence.

We set 17 clinicopathological factors, 30 preoperative blood test results, and 22 postoperative blood test results as explanatory variables. We then developed a prognostic model for predicting DFS, OS, and CSS using the eXtreme Gradient Boosting (XGBoost). To evaluate the discrimination of our AI prognostic model, time-dependent receiver operating characteristic (ROC) curves and area under curves (AUCs) for predicting DFS, OS, and CSS were calculated (Fig. 2). The AUCs of the AI prognostic model were better than that of p-Stage alone at all time points. These results indicate that the AI model greatly improved the prognostic accuracy compared with p-Stage alone by comprehensively analyzing a large number of variables.

### Identification of important explanatory variables for AI prognostic model

**Figure 3:**
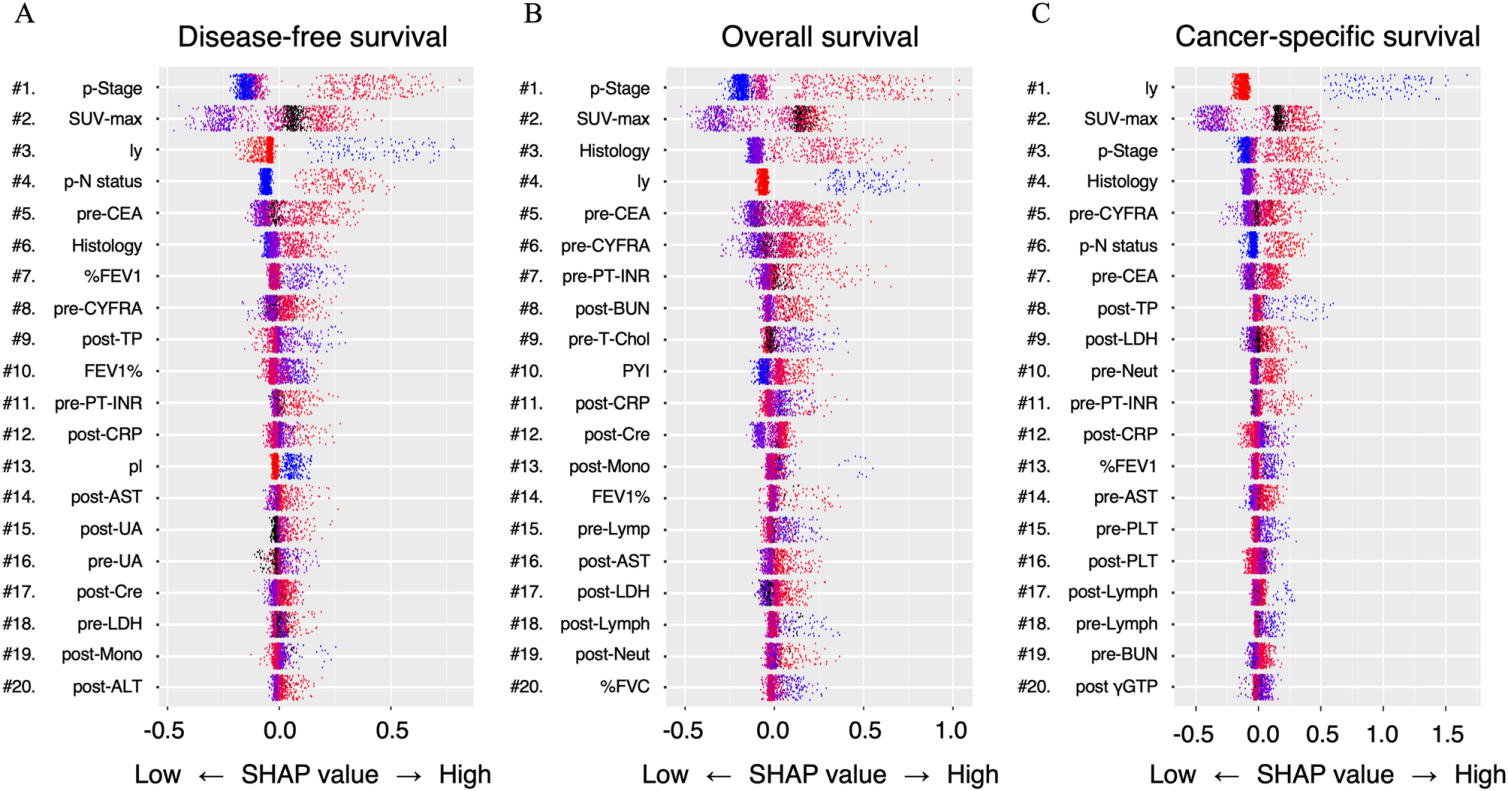
SHAP comprehensive plots. SHAP comprehensive plot with the top 20 explanatory variables that our AI prognostic model considered important for predicting prognosis of disease-free survival (**A**), overall survival (**B**), and cancer-specific survival (**C**). SHAP; SHapley Additive exPlanation, p-Stage; pathological stage, SUV; standard uptake value, ly; lymphatic invasion, p-N; pathological N, CEA; carcinoembryonic antigen, %FEV1.0; % forced expiratory volume in 1 s, CYFRA; cytokeratin-19 fragments, TP; total protein, FEV1.0%; forced expiratory volume in 1 s/ forced vital capacity ratio, PT-INR; prothrombin time-international normalized ratio, CRP; CRP; C-reactive protein, pl; pleural invasion, AST; aspartate aminotransferase, UA; urine acid, Cre; creatinine, LDH; lactate dehydrogenase, Mono; monocyte, ALT; alanine aminotransferase, BUN; blood urea nitrogen, T-Chol; total-cholesterol, PYI; pack year index, %FVC; % forced vital capacity, PLT; platelet, Lymph; lymphocyte, γ-GTP; γ-glutamyl transpeptidase.

We next analyzed the importance of each explanatory variable to DFS, OS, and CSS using the SHapley Additive exPlanation (SHAP) analysis to clarify which explanatory variables our AI prognostic model considered important in predicting prognosis (Fig. 3**)**. Each SHAP figure shows the SHAP comprehensive plot ^24–26^. In the SHAP comprehensive plot, explanatory variables that highly contributed to the prediction of survival in our AI prognostic model are arranged from the top. The rows correspond to explanatory variables, and each point in a row represents one patient. Red indicates a higher value of relevant variables, whereas blue indicates a lower value. Black dots indicate missing values. The horizontal axis represents the SHAP value, and the farther the dot is on the right, the higher the risk. The SHAP analysis showed that established prognostic factors such as tumor malignancy (p-Stage, pathological N status, histological type, pleural invasion, lymphatic invasion, standard uptake value (SUV)-max, carcinoembryonic antigen [CEA], and cytokeratin-19 fragments [CYFRA]), pulmonary function, and smoking history were still the top variables affecting the prognosis of NSCLC. In terms of blood test results, preoperative prothrombin time-international normalized ratio and postoperative CRP were identified as variables of high importance for DFS, OS, and CSS; postoperative total-protein, postoperative aspartate aminotransferase, postoperative creatinine, postoperative monocyte, preoperative lymphocyte, postoperative lymphocyte, and postoperative lactate dehydrogenase were identified as important variables in at least two among DFS, OS, and CSS.

### Calculation of predicted probability of survival based on AI prognostic model

**Figure 4:**
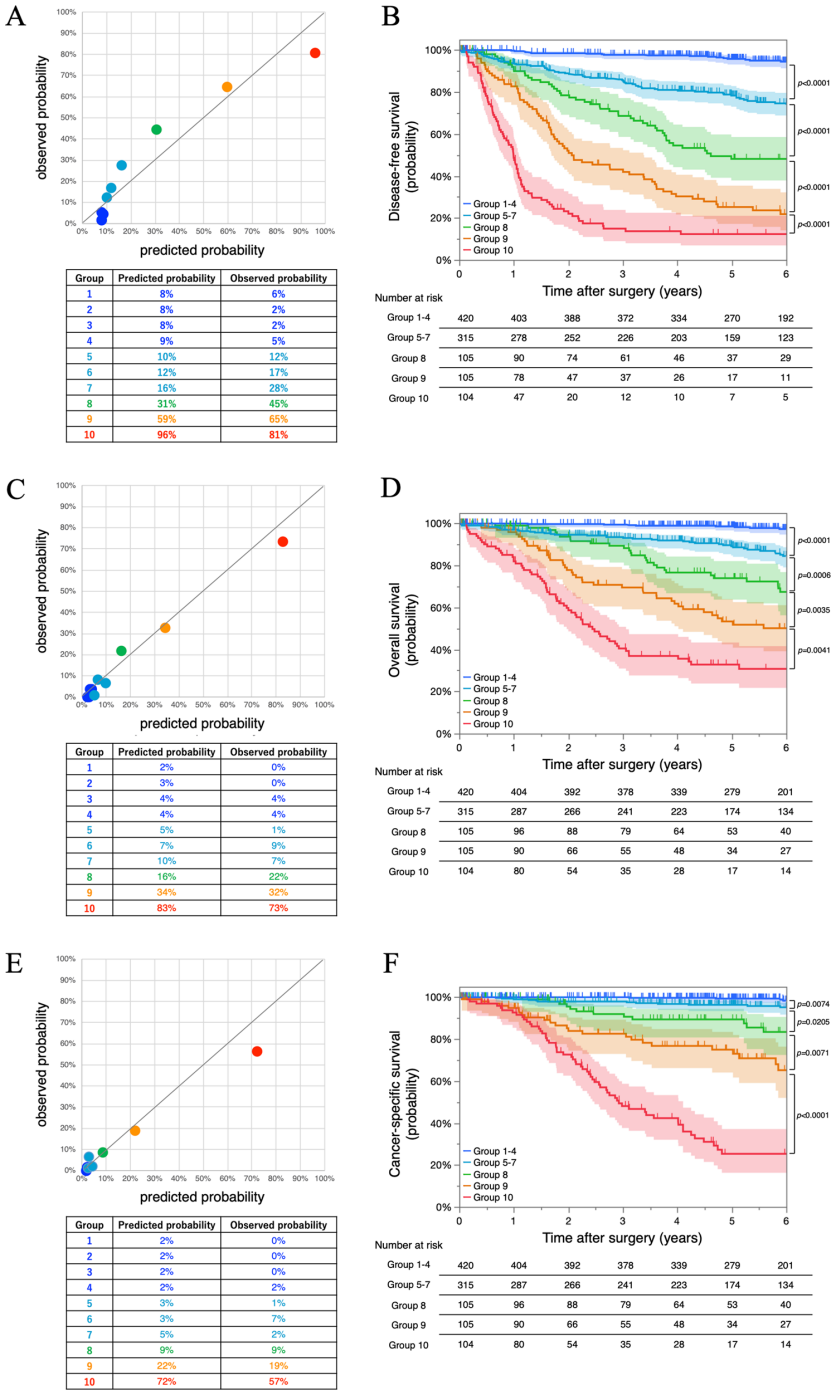
Calibration plots and survival curves based on the predicted probability of outcome events at 5 years from AI prognostic model. Calibration plots based on the predicted probability of outcome events at 5 years after surgery calculated by AI prognostic model for disease-free survival (**A**), overall survival (**C**), and cancer-specific survival (**E**). The survival curves for disease-free survival (**B**), overall survival (**D**), and cancer-specific survival (**F**) according to the predicted probability of outcome events at 5 years calculated by AI prognostic model. The 95% confidence intervals are shown via shading. AI; artificial intelligence.

To further evaluate the usefulness of the AI prognostic model, we calculated the predicted probability of outcome events at 5 years after surgery for each individual NSCLC patient based on our AI prognostic model. Patients were divided into 10 groups according to the predicted probability, and the calibration plots were generated for comparison of the observed probability (Fig. 4). The calibration plots showed a good match between the predicted and observed probability. Moreover, the survival curve created based on this predicted probability could stratify patients on prognosis (Fig. 4). The clinicopathological characteristics in the groups classified by the predicted probability for DFS, OS, and CSS are shown in Supplementary Table 6, 7 and 8.

## Discussion

We developed an AI prognostic model for surgically resected p-Stage I–IIIA NSCLC using machine learning to comprehensively analyze perioperative data. Our AI prognostic model showed good prediction accuracy, and the AUCs of the ROC curves in 5-year DFS, OS, and CSS were 0.890, 0.926, and 0.960, respectively. Furthermore, the predicted probability of outcome events at 5 years after surgery calculated by our AI prognostic model predicted the postoperative prognosis with high accuracy.

The application of AI has recently attracted much attention in the field of lung cancer, and several studies have used AI to predict the prognosis of lung cancer. Hosny et al. analyzed computed tomography (CT) images by deep learning in 1,194 patients with NSCLC to predict 2-year OS in radiotherapy and surgery groups; the results showed a prediction accuracy of AUC of 0.70 (95% confidence interval [CI], 0.63–0.78) in the radiotherapy group and AUC of 0.71 (95% CI, 0.60–0.82) in the surgery group^27^. Lu et al. analyzed CT images of 1,313 lung adenocarcinoma patients by machine learning and calculated intensity-skewness, which quantified the density distribution of tumor. The authors created ROC curves with DFS as the objective variable, and the results showed a prediction accuracy of AUC of 0.820–0.863^28^. She et al. used the Surveillance Epidemiology and End Results database and analyzed 1182 stage I–III NSCLC patients by 127 explanatory variables, including sex, age, marital status, tumor characteristics (location, size, histologic grade, histologic type, and TNM stage), and treatment details (surgical type). The prediction accuracy of the model for CSS was C-index of 0.739 (95% CI, 0.713–0.764)^29^. Kim et al. constructed a model for predicting DFS by analyzing CT images of 908 surgically resected lung adenocarcinoma patients using deep learning, and the prediction accuracy showed a C-index range of 0.71–0.78^30^. In most of the previous reports, CT images were analyzed by AI. We did not analyze CT images; instead, we used perioperative blood test results for analysis, which was not analyzed in previous studies. Although there were a few differences in the analysis methods, the prediction accuracy for 5-year DFS, OS, and CSS was AUC of 0.890, 0.926, and 0.960, respectively, which were not inferior to those of previous studies. On the basis of findings, we speculate that the use of a large amount of blood test results for predicting prognosis might have led to an accurate prognosis, reflecting not only the status of lung cancer, but also the nutritional status, immune status, and coexistence of other diseases such as renal disease, liver disease, and diabetes.

The prognosis of our cohort was relatively good, and the 5-year DFS, OS, and CSS were 71.0%, 82.8%, and 88.7%, respectively. The survival by p-Stage of our study cohort is almost the same as that of the Japanese large data cohort (18,973 patients), and these results are consistent with the general data on lung cancer in Japan^4^. Additionally, Kaplan–Meier survival curves showed that the survival of patients with p-Stages IIA and IIB was not well stratified in OS and CSS and reversed in DFS; we speculate this may be because of the limited number of patients with p-Stage IIA and IIB in our cohort.

In this study, XGBoost was selected as the algorithm for AI prognostic model. The advantages of using XGBoost compared with other AI tools are as follows. XGBoost has the advantage that missing values can be used directly as information^31^. This is not practical in the linear regression model, because it is necessary to delete the missing cases entirely or to fill in the missing values by assignment, but XGBoost is free from this problem. Moreover, a further advantage of decision tree models such as XGBoost is that interactions and nonlinearities can be automatically captured^31^. Furthermore, in SHAP analysis, only a decision tree-based model such as XGBoost can calculate accurate SHAP values^32^. Other methods (e.g., support vector machines, neural networks) are calculated by approximation, so accurate SHAP values are difficult. Therefore, we considered XGBoost to be suitable for constructing prognostic and predictive models such as in the present study.

A previous study of machine learning using blood test results attempted to diagnose lung cancer early by analyzing routine clinical and laboratory data of 6,505 patients in the NSCLC group and 189,597 patients in the control group using machine learning^22^. The results showed a good prediction accuracy of AUC of 0.86 and a diagnostic odds ratio of 12.3, suggesting that the analysis of blood test results might be highly valuable in lung cancer diagnosis and treatment^22^.

One of the features of the current study is that the data, including blood test results, for construction of the AI model was extracted from the electronic health record (EHR) system. The EHR system is used in many hospitals and has accumulated a huge amount of clinical data; these findings are expected to be applied to AI research in the future^33, 34^. Our research group previously developed a machine learning model for predicting the incidence of stroke-associated pneumonia by analyzing EHR systems^35^. Future research combining EHR systems and AI will provide many benefits in lung cancer treatment.

In addition to confirming the accuracy of prognosis prediction by machine learning, we evaluated the importance of each explanatory variable to the prognosis of NSCLC using SHAP values. When looking at each variable, established prognostic factors such as tumor malignancy (p-Stage, pathological N status, histological type, pleural invasion, lymphatic invasion, SUV-max, CEA, and CYFRA), pulmonary function, and smoking history were still the top factors affecting the prognosis of NSCLC; therefore, the validity of the evaluation by SHAP values is high. In AI-based research, “which variables” and “how much they contribute” are often black boxes that are difficult to understand. In our current study, SHAP analysis clarified “which variables” and “how much they contribute” to the AI prognostic model in predicting prognosis, which indicate that SHAP values may be very useful in the analysis of lung cancer in the future.

Histological type was revealed as an important factor of prognosis in our AI prognostic model. As shown in Supplementary Table 3, 4 and 5, the prognoses of adenocarcinoma in situ/minimally invasive adenocarcinoma/lepidic predominant adenocarcinoma and carcinoid were better than those of other histological types. In contrast, the prognoses of adenosquamous carcinoma, large cell neuroendocrine carcinoma, and pleomorphic carcinoma were worse than those of other histological types. Furthermore, the prognosis of micropapillary/solid predominant adenocarcinoma was as poor as that of squamous cell carcinoma and worse than that of acinar/papillary predominant adenocarcinoma. These results are consistent with previous findings of prognostic stratification by histological type and predominant adenocarcinoma^36, 37^. Our study showed that a more detailed analysis of histological type could improve the prognostic accuracy.

In terms of blood test results, tumor markers such as CEA and CYFRA still made significant contributions to prognosis. Factors used in immune-nutrition indices such as total cholesterol, CRP, neutrophil, lymphocyte, and monocyte also made a considerable contribution, and the results of this study supported previous reports that showed that the immune status of the host affects prognosis in NSCLC^7–16^. In addition, coagulation-related factors such as platelet, prothrombin time-international normalized ratio also made significant contributions. Cancer induces a hypercoagulable condition^38^, and considering the reports showing that coagulation-related factors are prognostic factors for NSCLC^39^, it is possible that coagulation-related factors reflect the activity of NSCLC. Additionally, factors reflecting renal and liver function, such as urea nitrogen, creatinine, and aspartate aminotransferase also contributed considerably to the prognosis of NSCLC. These factors might reflect poor patient heathy and influence survival.

We further calculated the predicted probability of outcome events at 5 years after surgery for each individual NSCLC patient on the basis of our AI prognostic model. The predicted probability was strongly associated with the observed probability and useful for predicting the prognosis of surgically resected NSCLC. Moreover, since the calculation of the predicted probability can be applied to other cohorts in addition to the cohort used in our study, we would like to further explore the feasibility of the AI prognostic model by performing external validation using cohorts from other institutions or temporal validation using a prospective cohort of cases. If we could further develop our AI prognostic model, we expect to be able to accurately predict the prognosis of individual patients with surgically resected NSCLC, which would be useful in determining treatment strategies, such as the selection of indications for postoperative adjuvant chemotherapy and differentiation of testing frequency according to prognosis.

This study has several limitations. This was a retrospective study with a single cohort from one institution and did not have validation cohorts. A prospective study of multiple cohorts at multiple institutions should be performed in the future to validate the usefulness of our AI prognostic model. The TNM classification 8th edition could not be used and the TNM classification 7th edition was used instead. The reason is that it was impossible to remeasure the diameter of the pathological invasive area of past cases. At our institution, the diameter of pathological invasive area has been evaluated since 2013; however, it was not possible to remeasure the diameter of pathological invasive area of cases before 2013. To conduct a large-scale analysis by including cases from 2003, we thus had to use the TNM classification 7th edition. We would like to conduct AI analysis research using the TNM 8th edition in the future. While our AI prognostic model was based on clinical information, pathological features, and blood test results, the AI prognostic model can be improved by adding more data such as information on underlying diseases, CT images, and pathological findings images. The information of driver oncogenes and PD-L1 expression is important for determining the prognosis of and treatment strategy for lung cancer patients; however, this information was unknown in many patients in our cohort, and therefore we could not include these factors in the analysis. In the XGBoost model, the formula itself is difficult to verify because the formula cannot be made explicit as in the old linear model. However, bootstrap validation was performed to verify the prediction accuracy, and the accuracy has been verified.

In conclusion, we developed an AI prognostic model for surgically resected NSCLC by comprehensively analyzing perioperative data using machine learning with XGBoost. The AI prognostic model using XGBoost showed good prediction accuracy and provided high accurate predictive probability of postoperative prognosis.

## Methods

### Study population

This retrospective observational study followed Declaration of Helsinki, and it was reviewed and approved by Kyushu University Institutional Review Board for Clinical Research on March 31st, 2021 (IRB No.2020-812), and a waiver of informed consent was obtained.

A total of 1,114 patients with p-Stage I–IIIA NSCLC underwent surgery between January 2003 and December 2016 at the Department of Surgery and Science, Graduate School of Medical Sciences, Kyushu University, Japan. Among these patients, 46, 9, and 10 patients were excluded because of preoperative treatment, incomplete resection, and second primary lung cancer, respectively. Finally, 1,049 patients were included for analysis.

### Dataset

We retrospectively obtained clinical information and follow-up data of patients by extracting data from the EHR system. The clinicopathological characteristics included age at surgery, sex, body mass index, smoking history (PYI), % forced vital capacity (%FVC), % forced expiratory volume in 1 s (%FEV1.0), FEV1.0% (FEV1.0/FVC ratio), SUV-max, surgical procedure, p-Stage, pathological T status, pathological N status, histological type, pleural invasion, vascular invasion, lymphatic invasion, and adjuvant chemotherapy. The PYI was calculated using the following formula: PYI = (cigarettes per day) x (smoking years)/20. The predictive FVC and predictive FEV1.0 were first calculated using formulae following the Clinical Pulmonary Functions Committee of the Japanese Respiratory Society; next, %FVC and %FEV1.0 were calculated by the following formulae: %FVC = observed FVC/predictive FVC and %FEV1 = observed FEV1.0/predictive FEV1.0, respectively.

We used blood test results used in routine practice and excluded data with a missing rate of more than 30% to make our selection. The preoperative blood test results, including total protein, albumin, total bilirubin, direct bilirubin, aspartate aminotransferase, alanine aminotransferase, alkaline phosphatase, γ-glutamyl transpeptidase, lactate dehydrogenase, urea nitrogen, creatinine, urine acid, sodium, potassium, chlorine, calcium, total-cholesterol, triglyceride, glucose, CRP, white blood cell, neutrophil, lymphocyte, monocyte, hemoglobin, platelet, prothrombin time-international normalized ratio, and activated partial thromboplastin time, were averaged over the one-month period before surgery. The blood tests were performed at various times, such as at the time of initial diagnosis and during preoperative hospitalization. Therefore, we decided to include data from a period of one month before surgery and to use mean values to minimize the effect of the timing of the tests. The preoperative levels of CEA and CYFRA were averaged over the three-month period before surgery. CEA and CYFRA were examined at the time of initial diagnosis in most cases; however, there were several cases in which CEA and CYFRA were examined more than one month before surgery because of preoperative examinations or patient convenience. Therefore, we decided to collect data on CEA and CYFRA for the 3-month period before surgery.

With regard to postoperative blood tests, considering that the postoperative hospitalization period is often 1–2 weeks, we decided to collect data for 2 weeks after surgery. The postoperative blood test results, including total protein, albumin, total bilirubin, aspartate aminotransferase, alanine aminotransferase, alkaline phosphatase, γ-glutamyl transpeptidase, lactate dehydrogenase, urea nitrogen, creatinine, urine acid, sodium, potassium, chlorine, calcium, CRP, white blood cell, neutrophil, lymphocyte, monocyte, hemoglobin, and platelet were averaged over the two-week period after surgery. To reduce the effect of variation in the postoperative blood test dates, the mean values were used. A summary of the blood test results of NSCLC patients is shown in Supplementary Table 2.

DFS was defined as the time between surgery and the date of recurrence or death from any cause. OS was defined as the time between surgery and the date of death from any cause. CSS was defined as the time between surgery and the date of death caused by NSCLC. Patients without an event were censored at the time of last follow-up.

### AI prognostic model

**Figure 5:**
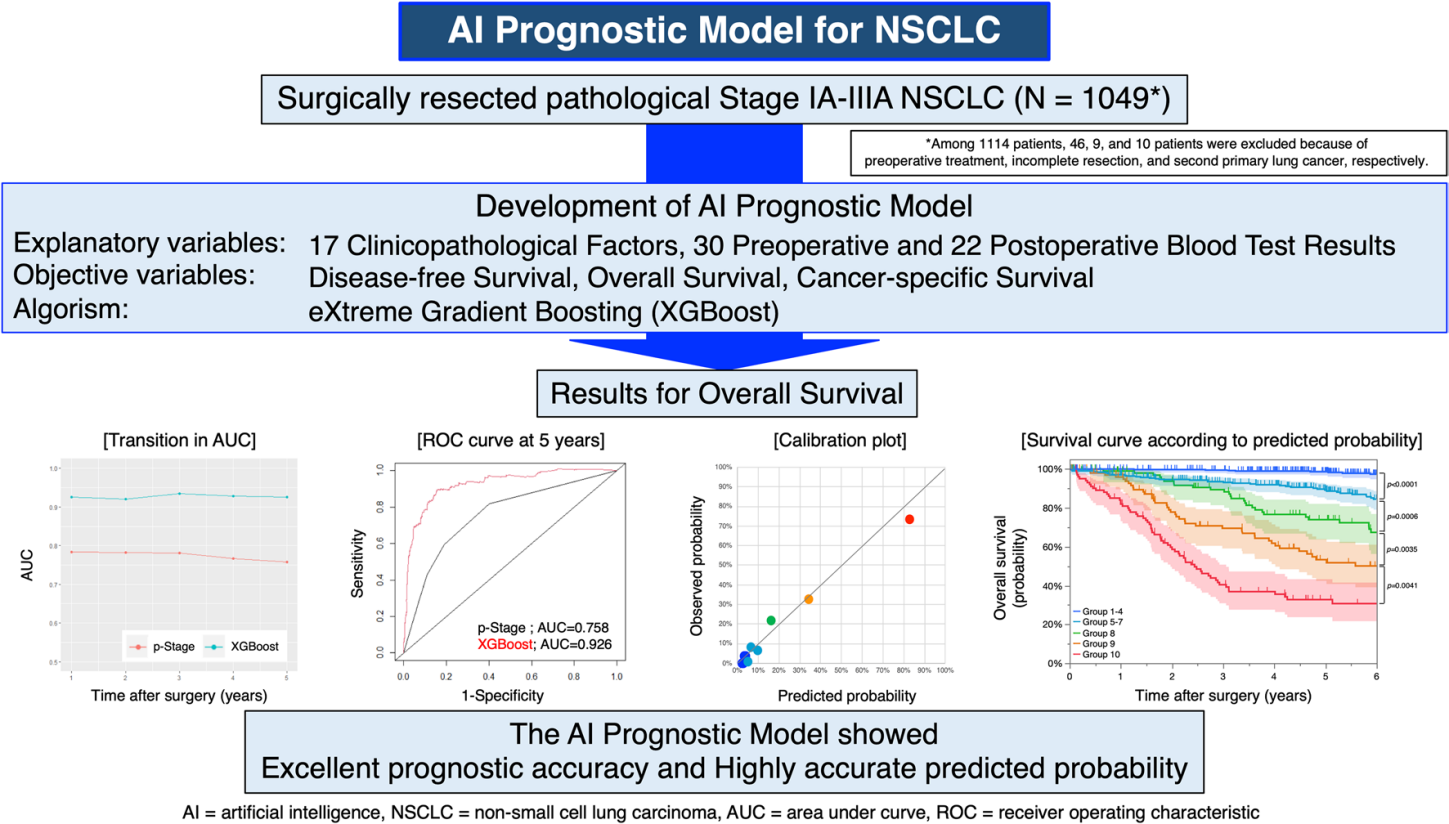
The study design.

The study design is summarized in Fig. 5. We set 17 clinicopathological factors, 30 preoperative blood test results, and 22 postoperative blood test results as explanatory variables. The DFS, OS, and CSS after surgery were set as objective variables. The XGBoost was used as the machine learning algorithm. The importance of each variable was analyzed using SHAP.

The XGBoost is a type of decision tree ensemble model that has desirable features such as capturing interactions and nonlinearities and handling missing values as they are^31^. We assumed non-linearity in predictors, such as blood test results, and clinical outcomes; therefore, XGBoost, which can automatically and accurately capture non-linearity, was chosen as the machine learning algorithm. To predict the risk for each patient, negative partial log-likelihood for Cox proportional hazards regression was used as the objective function of XGBoost. XGBoost can use missing values directly as information, and thus missing values were also analyzed as they are. To perform internal validation of our AI prognostic model, we performed bootstrap validations 20 times.

The SHAP is the most powerful technique for interpreting predictive models, and the Shapley value represents the contribution weight of each variable to the prediction model^24–26, 40^. The importance of objective variables is calculated, and the extraction of characteristic factors and their effects are expressed by a SHAP comprehensive plot. To evaluate the discriminative performance of the AI prognostic model, we created time-dependent ROC curves and AUCs for DFS, OS, and CSS^41^. The time-dependent ROC curves and AUCs of p-Stage were also created and compared with those of the AI prognostic model. As a calibration performance of outcome events at 5 years, the predicted probability was calculated based on the AI prognostic model and the calibration plot was created. The Kaplan–Meier curves were grouped based on the calibration plots and evaluated in each group.

### Statistical analysis

The Kaplan–Meier method with the log-rank test was used for plotting survival curves. The Cox proportional hazards model was used for univariable analysis, and the median value was set as the cutoff value of continuous variable. *p* < 0.05 indicated statistical significance. Statistical analyses were performed using JMP pro 14.0 software (SAS Institute) and R statistical software (http://www.R-project.org).

### Supplementary Information

Supplementary Table 1.

Supplementary Table 2.

Supplementary Table 3.

Supplementary Table 4.

Supplementary Table 5.

Supplementary Table 6.

Supplementary Table 7.

Supplementary Table 8.

## Data Availability

The data used to support the findings of our study are available from the corresponding author upon reasonable request.
