# Supplementary material for: Development of artificial intelligence prognostic model for surgically resected non-small cell lung cancer
Source: Sci Rep. 2023 Sep 21;13:15683. doi: 10.1038/s41598-023-42964-8 (PMC10514331; doi:10.1038/s41598-023-42964-8)
Supplement: Supplementary file 1 — Supplementary Table 1. [file 41598_2023_42964_MOESM1_ESM.docx]

**Supplementary Table 1. Clinicopathological characteristics of patients with NSCLC**

| Characteristics |  |  | No. of patients (%) | |  |
| --- | --- | --- | --- | --- | --- |
| Age, years | Median (range) |  | 69 | (23-89) |  |
| Sex | Female |  | 444 | (42.3%) |  |
|  | Male |  | 605 | (57.7%) |  |
| Height, cm | Median (range) |  | 160.0 | (131.0-182.8) |  |
| Weight, kg | Median (range) |  | 56.6 | (30.0-97.4) |  |
| Body mass index, kg/m^2^ | Median (range) |  | 22.4 | (14.0-34.3) |  |
| Pack year index | Median (range) |  | 20 | (0-300) |  |
| FVC, l | Median (range) |  | 3.01 | (1.19-5.59) |  |
| %FVC, % | Median (range) |  | 98.3 | (45.5-166.6) |  |
| FEV1.0, l/sec | Median (range) |  | 2.18 | (0.85-4.86) |  |
| %FEV1.0, % | Median (range) |  | 93.6 | (33.3-172.0) |  |
| FEV1.0%, % | Median (range) |  | 73.6 | (32.6-100.0) |  |
| SUV-max | Median (range) |  | 4 | (0-99) |  |
| Surgical procedure | Wedge resection |  | 144 | (13.7%) |  |
|  | Segmentectomy |  | 109 | (10.4%) |  |
|  | Lobectomy |  | 752 | (71.7%) |  |
|  | Bilobectomy |  | 24 | (2.3%) |  |
|  | Pneumonectomy |  | 20 | (1.9%) |  |
| pathological T status | 1a |  | 424 | (40.4%) |  |
|  | 1b |  | 189 | (18.0%) |  |
|  | 2a |  | 309 | (29.5%) |  |
|  | 2b |  | 52 | (5.0%) |  |
|  | 3 |  | 63 | (6.0%) |  |
|  | 4 |  | 12 | (1.1%) |  |
| pathological N status | 0 |  | 859 | (81.8%) |  |
|  | 1 |  | 96 | (9.2%) |  |
|  | 2 |  | 94 | (9.0%) |  |
| p-Stage | IA |  | 553 | (52.7%) |  |
|  | IB |  | 223 | (21.4%) |  |
|  | IIA |  | 100 | (9.5%) |  |
|  | IIB |  | 55 | (5.3%) |  |
|  | IIIA |  | 118 | (11.2%) |  |
| Histological type | AD-AIS/MIA/LEP |  | 151 | (14.4%) |  |
|  | AD-ACN/PAP |  | 574 | (54.7%) |  |
|  | AD-MIP/SOL |  | 55 | (5.2%) |  |
|  | AD-Others |  | 31 | (3.0%) |  |
|  | SQ |  | 189 | (18.0%) |  |
|  | ADSQ |  | 16 | (1.5%) |  |
|  | Carcinoid |  | 6 | (0.6%) |  |
|  | LCNEC |  | 23 | (2.2%) |  |
|  | Pleomorphic carcinoma |  | 4 | (0.4%) |  |
| Pleural invasion | Negative |  | 818 | (78.0%) |  |
|  | Positive |  | 231 | (22.0%) |  |
| Lymphatic invasion | Negative |  | 933 | (88.9 %) |  |
|  | Positive |  | 116 | (11.1%) |  |
| Vascular invasion | Negative |  | 774 | (73.8%) |  |
|  | Positive |  | 275 | (26.2%) |  |
| Adjuvant chemotherapy | None |  | 835 | (69.6%) |  |
|  | UFT |  | 106 | (10.1%) |  |
|  | TS-1 |  | 34 | (3.2%) |  |
|  | CDDP base |  | 57 | (5.4%) |  |
|  | CBDCA base |  | 17 | (1.6%) |  |

NSCLC; non-small cell lung carcinoma, FVC; forced vital capacity, FEV1; forced expiratory volume in 1 second, SUV; standard uptake value, p-Stage; pathological stage, AD; adenocarcinoma, AIS; adenocarcinoma in situ, MIA; minimally invasive adenocarcinoma, LEP; lepidic predominant adenocarcinoma, ACN; acinar predominant adenocarcinoma, PAP; papillary predominant adenocarcinoma, MIP; micropapillary predominant adenocarcinoma, SOL; solid predominant adenocarcinoma, SQ; squamous cell carcinoma, ADSQ; adenosquamous carcinoma, LCNEC; large cell neuroendocrine carcinoma, UFT; tegafur-uracil, CDDP; cisplatin, CBDCA; carboplatin
