# Supplementary material for: Development of artificial intelligence prognostic model for surgically resected non-small cell lung cancer
Source: Sci Rep. 2023 Sep 21;13:15683. doi: 10.1038/s41598-023-42964-8 (PMC10514331; doi:10.1038/s41598-023-42964-8)
Supplement: Supplementary file 2 — Supplementary Table 2. [file 41598_2023_42964_MOESM2_ESM.docx]

**Supplementary Table 2. Blood test results of patients with NSCLC**

| Blood test results |  |  | Median (range) | |
| --- | --- | --- | --- | --- |
| Preoperative |  |  |  |  |
| Total-protein | g/dL |  | 7.0 | (4.7-9.0) |
| Albumin | g/dL |  | 4.2 | (2.2-5.3) |
| Total-bilirubin | mg/dL |  | 0.6 | (0.2-4.3) |
| Direct-bilirubin | mg/dL |  | 0.1 | (0-2.7) |
| Aspartate aminotransferase | U/L |  | 21 | (8-157) |
| Alanine aminotransferase | U/L |  | 17 | (3-140) |
| Alkaline phosphatase | U/L |  | 226 | (78-888) |
| γ-glutamyl transpeptidase | U/L |  | 26 | (6-849) |
| Lactate dehydrogenase | U/L |  | 196 | (78-469) |
| Urea nitrogen | mg/dL |  | 15 | (6-85) |
| Creatinine | mg/dL |  | 0.74 | (0.41-10.89) |
| Urine acid | mg/dL |  | 5.3 | (2.2-11.7) |
| Sodium | mmol/L |  | 141 | (94-148) |
| Potassium | mmol/L |  | 4.2 | (2.5-5.6) |
| Chlorine | mmol/L |  | 105 | 70-114 |
| Calcium | mg/dL |  | 9.3 | 4.8-10.9 |
| Total-cholesterol | mg/dL |  | 197 | 91-308 |
| Triglyceride | mg/dL |  | 108 | 34-637 |
| Glucose | mg/dL |  | 125 | 62-311 |
| C-reactive protein | mg/dL |  | 0.10 | 0.01-16.82 |
| White blood cell | /μL |  | 5860 | 1790-22660 |
| Neutrophil | % |  | 62.1 | 18.4-93.7 |
| Lymphocyte | % |  | 27.9 | 4.1-67.0 |
| Monocyte | % |  | 5.5 | 2.0-18.5 |
| Hemoglobin | g/dL |  | 13.2 | 7.2-17.4 |
| Platelet | 10^3^/μL |  | 217 | 35-496 |
| Prothrombin time-international normalized ratio |  |  | 1.01 | 0.85-2.22 |
| Activated partial thromboplastin time | sec |  | 31.5 | 19.8-97.4 |
| Carcinoembryonic antigen | ng/mL |  | 3.0 | 0.2-175.9 |
| Cytokeratin-19 fragments | ng/mL |  | 2.0 | 0.6-51.6 |
| Postoperative |  |  |  |  |
| Total-protein | g/dL |  | 6.0 | 41.-7.8 |
| Albumin | g/dL |  | 3.3 | 1.9-4.5 |
| Total-bilirubin | mg/dL |  | 0.8 | 0.2-8.0 |
| Aspartate aminotransferase | U/L |  | 27 | 10-1938 |
| Alanine aminotransferase | U/L |  | 21 | 4-2049 |
| Alkaline phosphatase | U/L |  | 198 | 70-921 |
| γ-glutamyl transpeptidase | U/L |  | 28 | 6-613 |
| Lactate dehydrogenase | U/L |  | 213 | 112-6899 |
| Urea nitrogen | mg/dL |  | 14 | 6-100 |
| Creatinine | mg/dL |  | 0.70 | 0.38-9.74 |
| Urine acid | mg/dL |  | 4.1 | 1.2-11.0 |
| Sodium | mmol/L |  | 140 | 107-146 |
| Potassium | mmol/L |  | 4.1 | 3.0-5.5 |
| Chlorine | mmol/L |  | 104 | 82-112 |
| Calcium | mg/dL |  | 8.6 | 6.6-10.7 |
| C-reactive protein | mg/dL |  | 6.60 | 0.12-19.42 |
| White blood cell | /μL |  | 8780 | 3040-31400 |
| Neutrophil | % |  | 75.2 | 45.7-95.6 |
| Lymphocyte | % |  | 15.5 | 2.2-38.7 |
| Monocyte | % |  | 5.8 | 2.0-18.3 |
| Hemoglobin | g/dL |  | 11.8 | 7.0-15.7 |
| Platelet | 10^3^/μL |  | 228 | 41-533 |

NSCLC; non-small cell lung carcinoma,
