# Supplementary material for: Development of artificial intelligence prognostic model for surgically resected non-small cell lung cancer
Source: Sci Rep. 2023 Sep 21;13:15683. doi: 10.1038/s41598-023-42964-8 (PMC10514331; doi:10.1038/s41598-023-42964-8)
Supplement: Supplementary file 3 — Supplementary Table 3. [file 41598_2023_42964_MOESM3_ESM.docx]

**Supplementary Table 3. Univariable analysis for disease-free survival**

|  |  | Disease-free survival | | | | |
| --- | --- | --- | --- | --- | --- | --- |
|  |  | Univariable analysis | | | | |
| Characteristics |  | HR | 95% CI | | | *p* value |
| Age | < 69 years | Reference |  |  |  |  |
|  | ≥ 69 years | 1.46 | 1.163 | − | 1.839 | 0.0011 |
| Sex | Female | Reference |  |  |  |  |
|  | Male | 2.37 | 1.843 | − | 3.054 | <0.0001 |
| Body mass index | ≥ 22.4 kg/m2 | Reference |  |  |  |  |
|  | < 22.4 kg/m2 | 1.09 | 0.868 | − | 1.360 | 0.4688 |
| Pack year index | < 20 | Reference |  |  |  |  |
|  | ≥ 20 | 2.16 | 1.707 | − | 2.737 | <0.0001 |
| %FVC | ≥ 98.3% | Reference |  |  |  |  |
|  | < 98.3% | 1.56 | 1.242 | − | 1.962 | 0.0001 |
| %FEV1.0 | ≥ 93.6% | Reference |  |  |  |  |
|  | < 93.6% | 1.76 | 1.396 | − | 2.214 | <0.0001 |
| FEV1.0% | ≥ 73.6% | Reference |  |  |  |  |
|  | < 73.6% | 1.75 | 1.386 | − | 2.197 | <0.0001 |
| SUV-max | < 4 | Reference |  |  |  |  |
|  | ≥ 4 | 5.31 | 3.672 | − | 7.685 | <0.0001 |
| Surgical procedure | Wedge resection | Reference |  |  |  |  |
|  | Segmentectomy | 0.61 | 0.355 | − | 1.031 | 0.0646 |
|  | Lobectomy | 0.96 | 0.687 | − | 1.331 | 0.7916 |
|  | Bilobectomy | 2.34 | 1.255 | − | 4.361 | 0.0075 |
|  | Pneumonectomy | 2.46 | 1.295 | − | 4.683 | 0.0060 |
| p-Stage | IA | Reference |  |  |  |  |
|  | IB | 2.68 | 1.940 | − | 3.716 | <0.0001 |
|  | IIA | 6.08 | 4.287 | − | 8.616 | <0.0001 |
|  | IIB | 4.78 | 3.057 | − | 7.470 | <0.0001 |
|  | IIIA | 8.57 | 6.225 | − | 11.795 | <0.0001 |
| Histological type | AD-AIS/MIA/LEP | Reference |  |  |  |  |
|  | AD-ACN/PAP | 4.16 | 2.311 | − | 7.486 | <0.0001 |
|  | AD-MIP/SOL | 7.82 | 3.868 | − | 15.817 | <0.0001 |
|  | AD-Others | 3.03 | 1.137 | − | 8.084 | 0.0267 |
|  | SQ | 8.50 | 4.629 | − | 15.604 | <0.0001 |
|  | ADSQ | 9.60 | 3.920 | − | 23.498 | <0.0001 |
|  | Carcinoid | 2.01 | 0.262 | − | 15.501 | 0.5010 |
|  | LCNEC | 17.67 | 8.419 | − | 37.066 | <0.0001 |
|  | Pleomorphic carcinoma | 13.00 | 2.903 | − | 58.196 | 0.0008 |
| Pleural invasion | Negative | Reference |  |  |  |  |
|  | Positive | 3.65 | 2.903 | − | 4.587 | <0.0001 |
| Lymphatic invasion | Negative | Reference |  |  |  |  |
|  | Positive | 4.79 | 3.716 | − | 6.166 | <0.0001 |
| Vascular invasion | Negative | Reference |  |  |  |  |
|  | Positive | 3.14 | 2.504 | − | 3.934 | <0.0001 |
| pre-Albumin | ≥ 4.2 g/dL | Reference |  |  |  |  |
|  | < 4.2 g/dL | 2.00 | 1.586 | − | 2.517 | <0.0001 |
| pre-CRP | ≥ 0.10 mg/dL | Reference |  |  |  |  |
|  | < 0.10 mg/dL | 1.74 | 1.373 | − | 2.196 | <0.0001 |
| pre-Neutrophil | < 62.1% | Reference |  |  |  |  |
|  | ≥ 62.1% | 1.13 | 0.899 | − | 1.420 | 0.2962 |
| pre-Lymphocyte | ≥ 27.9% | Reference |  |  |  |  |
|  | < 27.9% | 1.27 | 1.014 | − | 1.601 | 0.0372 |
| pre-CEA | < 3.0 ng/mL | Reference |  |  |  |  |
|  | ≥ 3.0 ng/mL | 2.50 | 1.914 | − | 3.260 | <0.0001 |
| pre-CYFRA | < 2.0 ng/mL | Reference |  |  |  |  |
|  | ≥ 2.0 ng/mL | 2.98 | 2.258 | − | 3.929 | <0.0001 |

HR; hazard ratio, CI; confidence interval, FVC; forced vital capacity, FEV1; forced expiratory volume in 1 second, SUV; standard uptake value, p-Stage; pathological stage, AD; adenocarcinoma, AIS; adenocarcinoma in situ, MIA; minimally invasive adenocarcinoma, LEP; lepidic predominant adenocarcinoma, ACN; acinar predominant adenocarcinoma, PAP; papillary predominant adenocarcinoma, MIP; micropapillary predominant adenocarcinoma, SOL; solid predominant adenocarcinoma, SQ; squamous cell carcinoma, ADSQ; adenosquamous carcinoma, LCNEC; large cell neuroendocrine carcinoma
