# Supplementary material for: Development of artificial intelligence prognostic model for surgically resected non-small cell lung cancer
Source: Sci Rep. 2023 Sep 21;13:15683. doi: 10.1038/s41598-023-42964-8 (PMC10514331; doi:10.1038/s41598-023-42964-8)
Supplement: Supplementary file 4 — Supplementary Table 4. [file 41598_2023_42964_MOESM4_ESM.docx]

**Supplementary Table 4. Univariable analysis for overall survival**

|  |  | Overall survival | | | | |
| --- | --- | --- | --- | --- | --- | --- |
|  |  | Univariable analysis | | | | |
| Characteristics |  | HR | 95% CI | | | *p* value |
| Age | < 69 years | Reference |  |  |  |  |
|  | ≥ 69 years | 2.04 | 1.537 | − | 2.700 | <0.0001 |
| Sex | Female | Reference |  |  |  |  |
|  | Male | 2.59 | 1.906 | − | 3.521 | <0.0001 |
| Body mass index | ≥ 22.4 kg/m2 | Reference |  |  |  |  |
|  | < 22.4 kg/m2 | 1.10 | 0.838 | − | 1.433 | 0.5054 |
| Pack year index | < 20 | Reference |  |  |  |  |
|  | ≥ 20 | 2.79 | 2.082 | − | 3.743 | <0.0001 |
| %FVC | ≥ 98.3% | Reference |  |  |  |  |
|  | < 98.3% | 1.55 | 1.180 | − | 2.037 | 0.0016 |
| %FEV1.0 | ≥ 93.6% | Reference |  |  |  |  |
|  | < 93.6% | 1.79 | 1.357 | − | 2.360 | <0.0001 |
| FEV1.0% | ≥ 73.6% | Reference |  |  |  |  |
|  | < 73.6% | 1.68 | 1.276 | − | 2.210 | 0.0002 |
| SUV-max | < 4 | Reference |  |  |  |  |
|  | ≥ 4 | 4.37 | 2.816 | − | 6.791 | <0.0001 |
| Surgical procedure | Wedge resection | Reference |  |  |  |  |
|  | Segmentectomy | 0.65 | 0.350 | − | 1.209 | 0.1739 |
|  | Lobectomy | 0.88 | 0.595 | − | 1.305 | 0.5275 |
|  | Bilobectomy | 1.58 | 0.723 | − | 3.449 | 0.2516 |
|  | Pneumonectomy | 2.71 | 1.358 | − | 5.414 | 0.0047 |
| p-Stage | IA | Reference |  |  |  |  |
|  | IB | 2.37 | 1.619 | − | 3.477 | <0.0001 |
|  | IIA | 4.04 | 2.632 | − | 6.215 | <0.0001 |
|  | IIB | 5.15 | 3.147 | − | 8.426 | <0.0001 |
|  | IIIA | 5.95 | 4.084 | − | 8.663 | <0.0001 |
| Histological type | AD-AIS/MIA/LEP | Reference |  |  |  |  |
|  | AD-ACN/PAP | 2.54 | 1.392 | − | 4.626 | 0.0024 |
|  | AD-MIP/SOL | 4.40 | 2.033 | − | 9.530 | 0.0002 |
|  | AD-Others | 2.79 | 0.983 | − | 7.943 | 0.0539 |
|  | SQ | 7.22 | 3.897 | − | 13.385 | <0.0001 |
|  | ADSQ | 8.42 | 3.311 | − | 21.411 | <0.0001 |
|  | Carcinoid | * |  |  |  |  |
|  | LCNEC | 11.10 | 4.888 | − | 25.217 | <0.0001 |
|  | Pleomorphic carcinoma | 18.63 | 4.147 | − | 83.694 | 0.0001 |
| Pleural invasion | Negative | Reference |  |  |  |  |
|  | Positive | 2.78 | 2.110 | − | 3.668 | <0.0001 |
| Lymphatic invasion | Negative | Reference |  |  |  |  |
|  | Positive | 3.55 | 2.621 | − | 4.811 | <0.0001 |
| Vascular invasion | Negative | Reference |  |  |  |  |
|  | Positive | 2.77 | 2.115 | − | 3.625 | <0.0001 |
| pre-Albumin | ≥ 4.2 g/dL | Reference |  |  |  |  |
|  | < 4.2 g/dL | 2.00 | 1.520 | − | 2.635 | <0.0001 |
| pre-CRP | ≥ 0.10 mg/dL | Reference |  |  |  |  |
|  | < 0.10 mg/dL | 1.99 | 1.493 | − | 2.641 | <0.0001 |
| pre-Neutrophil | < 62.1% | Reference |  |  |  |  |
|  | ≥ 62.1% | 1.30 | 0.989 | − | 1.709 | 0.0598 |
| pre-Lymphocyte | ≥ 27.9% | Reference |  |  |  |  |
|  | < 27.9% | 1.62 | 1.231 | − | 2.131 | 0.0006 |
| pre-CEA | < 3.0 ng/mL | Reference |  |  |  |  |
|  | ≥ 3.0 ng/mL | 2.43 | 1.765 | − | 3.341 | <0.0001 |
| pre-CYFRA | < 2.0 ng/mL | Reference |  |  |  |  |
|  | ≥ 2.0 ng/mL | 4.24 | 2.961 | − | 6.075 | <0.0001 |

*Univariable analysis could not be performed due to no event.

HR; hazard ratio, CI; confidence interval, FVC; forced vital capacity, FEV1; forced expiratory volume in 1 second, SUV; standard uptake value, p-Stage; pathological stage, AD; adenocarcinoma, AIS; adenocarcinoma in situ, MIA; minimally invasive adenocarcinoma, LEP; lepidic predominant adenocarcinoma, ACN; acinar predominant adenocarcinoma, PAP; papillary predominant adenocarcinoma, MIP; micropapillary predominant adenocarcinoma, SOL; solid predominant adenocarcinoma, SQ; squamous cell carcinoma, ADSQ; adenosquamous carcinoma, LCNEC; large cell neuroendocrine carcinoma
