# Supplementary material for: Development of artificial intelligence prognostic model for surgically resected non-small cell lung cancer
Source: Sci Rep. 2023 Sep 21;13:15683. doi: 10.1038/s41598-023-42964-8 (PMC10514331; doi:10.1038/s41598-023-42964-8)
Supplement: Supplementary file 5 — Supplementary Table 5. [file 41598_2023_42964_MOESM5_ESM.docx]

**Supplementary Table 5. Univariable analysis for cancer-specific survival**

|  |  | Cancer-spesific survival | | | | |
| --- | --- | --- | --- | --- | --- | --- |
|  |  | Univariable analysis | | | | |
| Characteristics |  | HR | 95% CI | | | *p* value |
| Age | < 69 years | Reference |  |  |  |  |
|  | ≥ 69 years | 1.49 | 1.039 | − | 2.133 | 0.0303 |
| Sex | Female | Reference |  |  |  |  |
|  | Male | 1.84 | 1.261 | − | 2.684 | 0.0016 |
| Body mass index | ≥ 22.4 kg/m2 | Reference |  |  |  |  |
|  | < 22.4 kg/m2 | 1.05 | 0.737 | − | 1.496 | 0.7866 |
| Pack year index | < 20 | Reference |  |  |  |  |
|  | ≥ 20 | 1.82 | 1.269 | − | 2.622 | 0.0012 |
| %FVC | ≥ 98.3% | Reference |  |  |  |  |
|  | < 98.3% | 1.20 | 0.838 | − | 1.706 | 0.3231 |
| %FEV1.0 | ≥ 93.6% | Reference |  |  |  |  |
|  | < 93.6% | 1.25 | 0.876 | − | 1.784 | 0.2182 |
| FEV1.0% | ≥ 73.6% | Reference |  |  |  |  |
|  | < 73.6% | 1.63 | 1.134 | − | 2.335 | 0.0082 |
| SUV-max | < 4 | Reference |  |  |  |  |
|  | ≥ 4 | 11.39 | 4.916 | − | 26.383 | <0.0001 |
| Surgical procedure | Wedge resection | Reference |  |  |  |  |
|  | Segmentectomy | 0.79 | 0.329 | − | 1.916 | 0.6080 |
|  | Lobectomy | 1.23 | 0.685 | − | 2.194 | 0.4934 |
|  | Bilobectomy | 2.37 | 0.846 | − | 6.669 | 0.1006 |
|  | Pneumonectomy | 4.70 | 1.949 | − | 11.353 | 0.0006 |
| p-Stage | IA | Reference |  |  |  |  |
|  | IB | 3.57 | 2.095 | − | 6.087 | <0.0001 |
|  | IIA | 5.95 | 3.283 | − | 10.770 | <0.0001 |
|  | IIB | 6.73 | 3.366 | − | 13.472 | <0.0001 |
|  | IIIA | 9.90 | 5.897 | − | 16.628 | <0.0001 |
| Histological type | AD-AIS/MIA/LEP | Reference |  |  |  |  |
|  | AD-ACN/PAP | 2.46 | 1.119 | − | 5.385 | 0.0250 |
|  | AD-MIP/SOL | 4.30 | 1.557 | − | 11.872 | 0.0049 |
|  | AD-Others | 1.76 | 0.366 | − | 8.488 | 0.4803 |
|  | SQ | 6.57 | 2.922 | − | 14.754 | <0.0001 |
|  | ADSQ | 8.02 | 2.344 | − | 27.410 | 0.0009 |
|  | Carcinoid | * |  |  |  |  |
|  | LCNEC | 11.53 | 4.034 | − | 32.936 | <0.0001 |
|  | Pleomorphic carcinoma | 14.21 | 1.741 | − | 116.027 | 0.0132 |
| Pleural invasion | Negative | Reference |  |  |  |  |
|  | Positive | 3.31 | 2.314 | − | 4.743 | <0.0001 |
| Lymphatic invasion | Negative | Reference |  |  |  |  |
|  | Positive | 5.79 | 4.000 | − | 8.370 | <0.0001 |
| Vascular invasion | Negative | Reference |  |  |  |  |
|  | Positive | 3.01 | 2.112 | − | 4.290 | <0.0001 |
| pre-Albumin | ≥ 4.2 g/dL | Reference |  |  |  |  |
|  | < 4.2 g/dL | 1.84 | 1.283 | − | 2.633 | 0.0009 |
| pre-CRP | ≥ 0.10 mg/dL | Reference |  |  |  |  |
|  | < 0.10 mg/dL | 1.97 | 1.354 | − | 2.857 | 0.0004 |
| pre-Neutrophil | < 62.1% | Reference |  |  |  |  |
|  | ≥ 62.1% | 1.14 | 0.800 | − | 1.632 | 0.4639 |
| pre-Lymphocyte | ≥ 27.9% | Reference |  |  |  |  |
|  | < 27.9% | 1.53 | 1.069 | − | 2.196 | 0.0201 |
| pre-CEA | < 3.0 ng/mL | Reference |  |  |  |  |
|  | ≥ 3.0 ng/mL | 3.32 | 2.108 | − | 5.216 | <0.0001 |
| pre-CYFRA | < 2.0 ng/mL | Reference |  |  |  |  |
|  | ≥ 2.0 ng/mL | 3.60 | 2.262 | − | 5.726 | <0.0001 |

*Univariable analysis could not be performed due to no event.

HR; hazard ratio, CI; confidence interval, FVC; forced vital capacity, FEV1; forced expiratory volume in 1 second, SUV; standard uptake value, p-Stage; pathological stage, AD; adenocarcinoma, AIS; adenocarcinoma in situ, MIA; minimally invasive adenocarcinoma, LEP; lepidic predominant adenocarcinoma, ACN; acinar predominant adenocarcinoma, PAP; papillary predominant adenocarcinoma, MIP; micropapillary predominant adenocarcinoma, SOL; solid predominant adenocarcinoma, SQ; squamous cell carcinoma, ADSQ; adenosquamous carcinoma, LCNEC; large cell neuroendocrine carcinoma
