# Supplementary material for: Development of artificial intelligence prognostic model for surgically resected non-small cell lung cancer
Source: Sci Rep. 2023 Sep 21;13:15683. doi: 10.1038/s41598-023-42964-8 (PMC10514331; doi:10.1038/s41598-023-42964-8)
Supplement: Supplementary file 6 — Supplementary Table 6. [file 41598_2023_42964_MOESM6_ESM.docx]

**Supplementary Table 6. Clinicopathological characteristics of patients in groups by AI prognostic model of disease-free survival**

| Characteristics |  | group1−4 | N=420 | group5–7 | N=315 | group8 | N=105 | group9 | N=105 | group10 | N=104 |
| --- | --- | --- | --- | --- | --- | --- | --- | --- | --- | --- | --- |
| Age, years | Median (range) | 73 | (36-88) | 71 | (23-89) | 68 | (45-87) | 71 | (38-85) | 71 | (36-86) |
| Sex | Female | 239 | (56.9%) | 119 | (37.8%) | 34 | (32.4%) | 27 | (25.7%) | 25 | (24.0%) |
|  | Male | 181 | (43.1%) | 196 | (62.2%) | 71 | (67.6%) | 78 | (74.3%) | 79 | (76.0%) |
| Body mass index, kg/m^2^ | Median (range) | 22.3 | (14.0-32.5) | 22.6 | (14.9-32.2) | 21.9 | (14.4-34.3) | 22.8 | (15.9-31.0) | 21.8 | (16.3-33.7) |
| Pack year index | Median (range) | 0 | (0-135) | 25 | (0-160) | 38 | (0-165) | 40 | (0-220) | 39 | (0-300) |
| %FVC, % | Median (range) | 100.6 | (50.5-166.6) | 93.5 | (38.2-138.7) | 97.9 | (58.9-140.4) | 95.8 | (45.5-131.4) | 93.9 | (60-129.1) |
| %FEV1.0, % | Median (range) | 97.2 | (44.6-172.0) | 73.8 | (40.1-138.7) | 92.8 | (44.4-145.7) | 88.1 | (33.3-127.7) | 84.5 | (38.6-133.1) |
| FEV1.0%, % | Median (range) | 75.1 | (32.6-92.3) | 73.8 | (40.1-100) | 72.7 | (37.1-100) | 72.9 | (34.6-97.3) | 70.0 | (34.8-96.0) |
| SUV-max | Median (range) | 1.6 | (0-28.0) | 5.8 | (0-31.1) | 6.4 | (0-19.3) | 7.3 | (0-26.6) | 9.3 | (0-99.0) |
| Surgical procedure | Wedge resection | 80 | (19.0%) | 30 | (9.5%) | 9 | (8.6%) | 18 | (17.1%) | 7 | (6.7%) |
|  | Segmentectomy | 61 | (14.5%) | 28 | (8.9%) | 8 | (7.6%) | 5 | (4.8%) | 7 | (6.7%) |
|  | Lobectomy | 276 | (65.7%) | 248 | (78.7%) | 79 | (75.2%) | 76 | (72.4%) | 73 | (70.2%) |
|  | Bilobectomy | 1 | (0.2%) | 5 | (1.6%) | 7 | (6.7%) | 3 | (2.9%) | 8 | (7.7%) |
|  | Pneumonectomy | 2 | (0.5%) | 4 | (1.3%) | 2 | (1.9%) | 3 | (2.9%) | 9 | (8.7%) |
| p-Stage | IA | 394 | (93.8%) | 85 | (27.0%) | 40 | (38.1%) | 26 | (24.8%) | 8 | (7.7%) |
|  | IB | 19 | (4.5%) | 150 | (47.6%) | 22 | (21.0%) | 26 | (24.8%) | 6 | (5.8%) |
|  | IIA | 3 | (0.7%) | 36 | (11.4%) | 18 | (17.1%) | 15 | (14.3%) | 28 | (26.9%) |
|  | IIB | 3 | (0.7%) | 15 | (4.8%) | 6 | (5.7%) | 13 | (12.4%) | 18 | (17.3%) |
|  | IIIA | 1 | (0.2%) | 29 | (9.2%) | 19 | (18.1%) | 25 | (23.8%) | 44 | (42.3%) |
| Histological type | AD-AIS/MIA/LEP | 111 | (26.4%) | 25 | (7.9%) | 10 | (9.5%) | 4 | (3.8%) | 1 | (1.0%) |
|  | AD-ACN/PAP | 250 | (59.5%) | 175 | (55.6%) | 54 | (51.4%) | 45 | (42.9%) | 50 | (48.5%) |
|  | AD-MIP/SOL | 10 | (2.4%) | 20 | (6.3%) | 6 | (5.7%) | 10 | (9.5%) | 9 | (8.7%) |
|  | AD-Others | 12 | (2.9%) | 12 | (3.8%) | 2 | (1.9%) | 3 | (2.9%) | 2 | (1.9%) |
|  | SQ | 32 | (7.6%) | 70 | (22.2%) | 22 | (21.0%) | 34 | (32.4%) | 31 | (30.1%) |
|  | ADSQ | 0 | (0.0%) | 5 | (1.6%) | 4 | (3.8%) | 4 | (3.8%) | 3 | (2.9%) |
|  | Carcinoid | 5 | (1.2%) | 0 | (0.0%) | 0 | (0.0%) | 0 | (0.0%) | 0 | (0.0%) |
|  | LCNEC | 0 | (0.0%) | 7 | (2.2%) | 6 | (5.7%) | 5 | (4.8%) | 5 | (4.9%) |
|  | Pleomorphic carcinoma | 0 | (0.0%) | 1 | (0.3%) | 1 | (1.0%) | 0 | (0.0%) | 2 | (1.9%) |
| Pleural invasion | Negative | 418 | (99.5%) | 214 | (67.9%) | 80 | (76.2%) | 58 | (55.2%) | 48 | (46.2%) |
|  | Positive | 2 | (0.5%) | 101 | (32.1%) | 25 | (23.8%) | 47 | (44.8%) | 56 | (53.8%) |
| Lymphatic invasion | Negative | 413 | (98.3%) | 282 | (89.5%) | 95 | (90.5%) | 92 | (87.6%) | 51 | (49.0%) |
|  | Positive | 7 | (1.7%) | 33 | (10.5%) | 10 | (9.5%) | 13 | (12.4%) | 53 | (51.0%) |
| Vascular invasion | Negative | 410 | (97.6%) | 200 | (63.5%) | 62 | (59.0%) | 60 | (57.1%) | 42 | (40.4%) |
|  | Positive | 10 | (2.4%) | 115 | (36.5%) | 43 | (41.0%) | 45 | (42.9%) | 62 | (59.6%) |
| pre-Albumin | Median (range) | 4.3 | (2.6-5.3) | 4.1 | (2.7-5.1) | 4.2 | (3.3-4.9) | 4.1 | (2.6-4.8) | 4.0 | (2.2-4.7) |
| pre-CRP | Median (range) | 0.06 | (0.01-9.66) | 0.09 | (0.01-10.54) | 0.12 | (0.01-8.44) | 0.21 | (0.01-6.61) | 0.32 | (0.01-16.82) |
| pre-Neutrophil | Median (range) | 61.2 | (27.0-87.3) | 61.7 | (30.6-84.0) | 63.3 | (18.4-87.3) | 62.7 | (43.2-87.8) | 66.5 | (38.0-93.7) |
| pre-Lymphocyte | Median (range) | 28.7 | (7.0-67.0) | 28.8 | (8.7-57.9) | 27.2 | (6.9-60.4) | 26.5 | (6.2-46.4) | 24.4 | (4.1-50.6) |
| pre-CEA | Median (range) | 2.2 | (0.2-14.0) | 3.5 | (0.5-113.2) | 4.0 | (0.5-121) | 4.2 | (0.6-175.9) | 5.1 | (0.9-129.8) |
| pre-CYFRA | Median (range) | 1.6 | (0.7-9.1) | 2.1 | (0.6-33.8) | 2.2 | (0.6-14.6) | 2.7 | (0.7-7.8) | 3.0 | (0.8-51.6) |

AI; artificial intelligence, FVC; forced vital capacity, FEV1; forced expiratory volume in 1 second, SUV; standard uptake value, p-Stage; pathological stage, AD; adenocarcinoma, AIS; adenocarcinoma in situ, MIA; minimally invasive adenocarcinoma, LEP; lepidic predominant adenocarcinoma, ACN; acinar predominant adenocarcinoma, PAP; papillary predominant adenocarcinoma, MIP; micropapillary predominant adenocarcinoma, SOL; solid predominant adenocarcinoma, SQ; squamous cell carcinoma, ADSQ; adenosquamous carcinoma, LCNEC; large cell neuroendocrine carcinoma, CRP; C-reactive protein, CEA; carcinoembryonic antigen, CYFRA; cytokeratin-19 fragments
