# Supplementary material for: Development of artificial intelligence prognostic model for surgically resected non-small cell lung cancer
Source: Sci Rep. 2023 Sep 21;13:15683. doi: 10.1038/s41598-023-42964-8 (PMC10514331; doi:10.1038/s41598-023-42964-8)
Supplement: Supplementary file 8 — Supplementary Table 8. [file 41598_2023_42964_MOESM8_ESM.docx]

**Supplementary Table 8. Clinicopathological characteristics of patients in groups by AI prognostic model of cancer-specific survival**

| Characteristics |  | group1−4 | N=420 | group5–7 | N=315 | group8 | N=105 | group9 | N=105 | group10 | N=104 |
| --- | --- | --- | --- | --- | --- | --- | --- | --- | --- | --- | --- |
| Age, years | Median (range) | 68 | (23-88) | 70 | (38-88) | 71 | (29-89) | 72 | (47-86) | 71 | (36-86) |
| Sex | Female | 228 | (54.3%) | 124 | (39.4%) | 36 | (34.3%) | 29 | (27.6%) | 27 | (26.0%) |
|  | Male | 192 | (45.7%) | 191 | (60.6%) | 69 | (65.7%) | 76 | (72.4%) | 77 | (74.0%) |
| Body mass index, kg/m^2^ | Median (range) | 22.4 | (15.2-33.7) | 22.6 | (14.0-34.3) | 22.1 | (14.9-32.5) | 22.4 | (14.4-30.8) | 21.7 | (15.9-31.2) |
| Pack year index | Median (range) | 0 | (0-220) | 24 | (0-165) | 25 | (0-300) | 41 | (0-150) | 39 | (0-196) |
| %FVC, % | Median (range) | 99.5 | (51.5-166.6) | 97.8 | (50.5-150.6) | 99.5 | (55.6-148.2) | 98.3 | (64.3-134.5) | 94.3 | (45.5-124.8) |
| %FEV1.0, % | Median (range) | 96.6 | (44.0-172.0) | 90.9 | (38.7-148.8) | 95.3 | (38.2-128.6) | 91.0 | (47.8-136.1) | 88.9 | (33.3-133.1) |
| FEV1.0%, % | Median (range) | 74.8 | (46.0-100) | 73.5 | (37.1-97.6) | 74.5 | (32.9-97.3) | 72.5 | (32.6-95.9) | 72.6 | (34.8-96.0) |
| SUV-max | Median (range) | 1.7 | (0-16.9) | 7.3 | (0-31.1) | 7.7 | (0-99.0) | 9.2 | (2.1-27.7) | 9.7 | (0-30.6) |
| Surgical procedure | Wedge resection | 81 | (19.3%) | 26 | (8.3%) | 18 | (17.1%) | 12 | (11.4%) | 7 | (6.7%) |
|  | Segmentectomy | 57 | (13.6%) | 23 | (7.3%) | 11 | (10.5%) | 11 | (10.5%) | 7 | (6.7%) |
|  | Lobectomy | 280 | (66.7%) | 254 | (80.6%) | 68 | (64.8%) | 74 | (70.5%) | 76 | (73.1%) |
|  | Bilobectomy | 2 | (0.5%) | 8 | (2.5%) | 4 | (3.8%) | 5 | (4.8%) | 5 | (4.8%) |
|  | Pneumonectomy | 0 | (0.0%) | 4 | (1.3%) | 4 | (3.8%) | 3 | (2.9%) | 9 | (8.7%) |
| p-Stage | IA | 339 | (80.7%) | 132 | (41.9%) | 33 | (31.4%) | 34 | (32.4%) | 15 | (14.4%) |
|  | IB | 57 | (13.6%) | 96 | (30.5%) | 31 | (29.5%) | 25 | (23.8%) | 14 | (13.5%) |
|  | IIA | 16 | (3.8%) | 34 | (10.8%) | 10 | (9.5%) | 20 | (19.0%) | 20 | (19.2%) |
|  | IIB | 3 | (0.7%) | 17 | (5.4%) | 9 | (8.6%) | 10 | (9.5%) | 16 | (15.4%) |
|  | IIIA | 5 | (1.2%) | 36 | (11.4%) | 22 | (21.0%) | 16 | (15.2%) | 39 | (37.5%) |
| Histological type | AD-AIS/MIA/LEP | 95 | (22.6%) | 42 | (13.3%) | 5 | (4.8%) | 6 | (5.7%) | 3 | (2.9%) |
|  | AD-ACN/PAP | 273 | (65.0%) | 156 | (49.5%) | 67 | (63.8%) | 42 | (40.0%) | 36 | (34.6%) |
|  | AD-MIP/SOL | 11 | (2.6%) | 24 | (7.6%) | 8 | (7.6%) | 6 | (5.7%) | 6 | (5.8%) |
|  | AD-Others | 18 | (4.3%) | 9 | (2.9%) | 1 | (1.0%) | 2 | (1.9%) | 1 | (1.0%) |
|  | SQ | 18 | (4.3%) | 66 | (21.0%) | 20 | (19.0%) | 41 | (39.0%) | 44 | (42.3%) |
|  | ADSQ | 1 | (0.2%) | 7 | (2.2%) | 0 | (0.0%) | 4 | (3.8%) | 4 | (3.8%) |
|  | Carcinoid | 2 | (0.5%) | 4 | (1.3%) | 0 | (0.0%) | 0 | (0.0%) | 0 | (0.0%) |
|  | LCNEC | 2 | (0.5%) | 7 | (2.2%) | 4 | (3.8%) | 3 | (2.9%) | 7 | (6.7%) |
|  | Pleomorphic carcinoma | 0 | (0.0%) | 0 | (0.0%) | 0 | (0.0%) | 1 | (1.0%) | 3 | (2.9%) |
| Pleural invasion | Negative | 402 | (95.7%) | 228 | (72.4%) | 70 | (66.7%) | 66 | (62.9%) | 52 | (50.0%) |
|  | Positive | 18 | (4.3%) | 87 | (27.6%) | 35 | (33.3%) | 39 | (37.1%) | 52 | (50.0%) |
| Lymphatic invasion | Negative | 407 | (96.9%) | 288 | (91.4%) | 85 | (81.0%) | 95 | (90.5%) | 58 | (55.8%) |
|  | Positive | 13 | (3.1%) | 27 | (8.6%) | 20 | (19.0%) | 10 | (9.5%) | 46 | (44.2%) |
| Vascular invasion | Negative | 389 | (92.6%) | 215 | (68.3%) | 65 | (61.9%) | 57 | (54.3%) | 48 | (46.2%) |
|  | Positive | 31 | (7.4%) | 100 | (31.7%) | 40 | (38.1%) | 48 | (45.7%) | 56 | (53.8%) |
| pre-Albumin | Median (range) | 4.2 | (3.1-5.3) | 4.2 | (2.7-4.9) | 4.1 | (2.6-5.1) | 4.1 | (2.6-4.8) | 4.0 | (2.2-4.7) |
| pre-CRP | Median (range) | 0.06 | (0.01-5.18) | 0.12 | (0.01-5.28) | 0.16 | (0.01-10.54) | 0.19 | (0.01-7.94) | 0.19 | (0.01-16.82) |
| pre-Neutrophil | Median (range) | 61.3 | (18.4-83.9) | 61.8 | (27.0-93.7) | 65.0 | (39.3-87.3) | 64.6 | (38.7-87.8) | 61.6 | (28.6-87.3) |
| pre-Lymphocyte | Median (range) | 28.6 | (8.9-45.8) | 28.9 | (4.1-67.0) | 25.5 | (7.0-49.1) | 26.5 | (6.2-56.4) | 26.8 | (6.9-60.4) |
| pre-CEA | Median (range) | 2.3 | (0.5-35.3) | 3.6 | (0.2-121.0) | 3.1 | (0.6-129.8) | 3.7 | (0.6-26.1) | 4.5 | (0.8-175.8) |
| pre-CYFRA | Median (range) | 1.7 | (0.6-8.0) | 1.9 | (0.6-30.8) | 2.4 | (0.7-51.6) | 2.7 | (1.1-33.8) | 3.3 | (0.8-14.6) |

AI; artificial intelligence, FVC; forced vital capacity, FEV1; forced expiratory volume in 1 second, SUV; standard uptake value, p-Stage; pathological stage, AD; adenocarcinoma, AIS; adenocarcinoma in situ, MIA; minimally invasive adenocarcinoma, LEP; lepidic predominant adenocarcinoma, ACN; acinar predominant adenocarcinoma, PAP; papillary predominant adenocarcinoma, MIP; micropapillary predominant adenocarcinoma, SOL; solid predominant adenocarcinoma, SQ; squamous cell carcinoma, ADSQ; adenosquamous carcinoma, LCNEC; large cell neuroendocrine carcinoma, CRP; C-reactive protein, CEA; carcinoembryonic antigen, CYFRA; cytokeratin-19 fragments
